# Complete Genome Sequence of Rnf- and Cytochrome-Containing Autotrophic Acetogen *Clostridium aceticum* DSM 1496

**DOI:** 10.1128/genomeA.00786-15

**Published:** 2015-07-16

**Authors:** Anja Poehlein, Frank R. Bengelsdorf, Bettina Schiel-Bengelsdorf, Gerhard Gottschalk, Rolf Daniel, Peter Dürre

**Affiliations:** a Genomic and Applied Microbiology & Göttingen Genomics Laboratory, Georg-August University Göttingen, Göttingen, Germany; b Institut für Mikrobiologie und Biotechnologie, Universität Ulm, Ulm, Germany

## Abstract

Here, we report the closed genome sequence of *Clostridium aceticum*, an Rnf- and cytochrome-containing autotrophic acetogen that is able to convert CO_2_ and H_2_ to acetate using the Wood-Ljungdahl pathway. The genome consists of a circular chromosome (4.2 Mbp) and a small circular plasmid (5.7 kbp).

## GENOME ANNOUNCEMENT

*Clostridium aceticum*, an anaerobic and endospore-forming organism, was the first bacterial isolate described to be able to form acetate autotrophically from a CO_2_ + H_2_ gas mixture. This organism is also able to grow heterotrophically on sugars, organic acids, and alcohols. *C. aceticum* was originally isolated from sludge from a town canal in Wageningen in 1936 (1–3), thought to be lost during World War II, and was reisolated from a spore preparation by one of us (G.G.) in 1979 (4). Chromosomal DNA was isolated using the MasterPure complete DNA purification kit (Epicentre, Madison, WI, USA). 454 shotgun and paired-end libraries and Illumina shotgun libraries were generated from the extracted DNA according to the protocol of the manufacturer. Sequencing was performed using a 454 GS-FLX system (Titanium GS70 chemistry; Roche Life Science, Mannheim, Germany) and a Genome Analyzer II (Illumina, San Diego, CA) resulting in 374,679 total 454 shotgun reads containing 182,232 paired reads with an average pair distance of 5.7 kb and a pair distance deviation of 1.4 kb and 2,280,716 Illumina 112-bp paired-end reads. Mira 3.4 (5) software and Roche Newbler Assembly 2.3 software were used to perform the hybrid *de novo* assembly resulting in 77 contigs within 24 scaffolds with an average coverage of 76.97-fold. The remaining gaps were closed by PCR-based techniques and primer walking with Sanger sequencing of the products using BigDye 3.0 chemistry and an ABI3730XL capillary sequencer (Applied Biosystems, Life Technology GmbH, Darmstadt, Germany). The closed genome of *C. aceticum* consists of a circular chromosome (4.2 Mbp) and a small circular plasmid (5.7 kbp) with an overall G+C content of 35.3%. Automatic gene prediction was performed by using the software tool Prodigal (6). Genes coding for rRNA and tRNA were identified using RNAmmer (7) and tRNAscan (8), respectively. The Integrated Microbial Genomes-Expert Review (IMG-ER) system (9) was used for automatic annotation, which was subsequently manually curated by using the Swiss-Prot, TrEMBL, and InterPro databases (10). The genome harbored 6 rRNA cluster, 74 tRNA genes, 3,181 protein-coding genes with predicted functions, and 743 genes coding for hypothetical proteins. Genes coding for enzymes involved in the methyl and carbonyl branch of the Wood-Ljungdahl pathway are organized in one large gene cluster together with P2 and P3 proteins of the glycine decarboxylase (11). This cluster showed the same arrangement as identified in other autotrophic clostridia such as *C. ljungdahlii* (12) and *C. autoethanogenum* (13). Genome analysis also revealed the presence of genes encoding the Rnf complex and all genes necessary for cytochrome synthesis, but no genes coding for quinone biosynthesis are present. *C. aceticum* is also able to grow heterotrophically on fructose, fumarate or malate, but might also be able to use glycine or betaine as energy and carbon source, as gene clusters coding for a glycine and betaine reductase comparable to those identified in *Eubacterium acidaminophilum* (14) and *Sporomusa ovata* (15) are encoded.

### Nucleotide sequence accession numbers.

This whole-genome shotgun project has been deposited at DDBJ/EMBL/GenBank under the accession numbers CP009687 and CP009688.
